# Diagnostic accuracy of DXA compared to conventional spine radiographs for the detection of vertebral fractures in children

**DOI:** 10.1007/s00330-016-4556-3

**Published:** 2016-09-21

**Authors:** E. Adiotomre, L. Summers, A. Allison, S. J. Walters, M. Digby, P. Broadley, I. Lang, G. Morrison, N. Bishop, P. Arundel, A. C. Offiah

**Affiliations:** 1 Radiology Department, Sheffield Teaching Hospitals NHS Foundation Trust, Glossop Rd, Sheffield, South Yorkshire S10 2JF UK; 2 Radiology Department, Sheffield Children’s Hospital NHS Foundation Trust, Western Bank, Sheffield, South Yorkshire S10 2TH UK; 3 Sheffield Medical School, University of Sheffield, Beech Hill Rd, Sheffield, South Yorkshire S10 2RX UK; 4 School of Health and Related Research, University of Sheffield, 30 Regent St, Sheffield, South Yorkshire S1 4DA UK; 5 Medical Physics, Sheffield Teaching Hospitals NHS Foundation Trust, Glossop Rd, Sheffield, South Yorkshire S10 2JF UK; 6 Academic Unit of Child Health, University of Sheffield, Western Bank, Sheffield, South Yorkshire S10 2TH UK

**Keywords:** Spinal fractures, Osteoporosis, Interobserver variability, Dual energy x-ray absorptiometry, Radiography

## Abstract

**Objectives:**

In children, radiography is performed to diagnose vertebral fractures and dual energy x-ray absorptiometry (DXA) to assess bone density. In adults, DXA assesses both. We aimed to establish whether DXA can replace spine radiographs in assessment of paediatric vertebral fractures.

**Methods:**

Prospectively, lateral spine radiographs and lateral spine DXA of 250 children performed on the same day were independently scored by three radiologists using the simplified algorithm-based qualitative technique and blinded to results of the other modality. Consensus radiograph read and second read of 100 random images were performed. Diagnostic accuracy, inter/intraobserver and intermodality agreements, patient/carer experience and radiation dose were assessed.

**Results:**

Average sensitivity and specificity (95 % confidence interval) in diagnosing one or more vertebral fractures requiring treatment was 70 % (58–82 %) and 97 % (94–100 %) respectively for DXA and 74 % (55–93 %) and 96 % (95–98 %) for radiographs. Fleiss’ kappa for interobserver and average kappa for intraobserver reliability were 0.371 and 0.631 respectively for DXA and 0.418 and 0.621 for radiographs. Average effective dose was 41.9 μSv for DXA and 232.7 μSv for radiographs. Image quality was similar.

**Conclusion:**

Given comparable image quality and non-inferior diagnostic accuracy, lateral spine DXA should replace conventional radiographs for assessment of vertebral fractures in children.

***Key Points*:**

• *Vertebral fracture diagnostic accuracy of lateral spine DXA is non*-*inferior to radiographs*.

• *The rate of unreadable vertebrae for DXA is lower than for radiographs*.

• *Effective dose of DXA is significantly lower than radiographs*.

• *Children prefer DXA to radiographs*.

• *Given the above*, *DXA should replace radiographs for paediatric vertebral fracture assessment*.

## Introduction

Radiation exposure is directly associated with cancer risk [1–3]. The earlier the radiation exposure, the higher the risk of radiation-induced cancer [4, 5]. Children have a higher mitotic rate and therefore increased susceptibility to radiation and a longer lifespan to accumulate dose and manifest radiation-induced cancer [4, 6]. Repeated spine radiographs in adolescent scoliosis [7] and fluoroscopy in tuberculosis [8] are associated with increased risk of breast cancer. There is no minimum dose threshold at which radiation does not have a cancer risk but the dose response is linear for solid cancers and linear-quadratic for leukaemia [4, 5]. The Committee on Biological Effects on Ionizing Radiation VII lifetime risk model suggests that an increase of 100 mSv above background radiation could cause 1 cancer per 100 people [9]. The typical effective dose (ED) of one chest radiograph in a 10-year-old child is 0.006 mSv [5]. A study on cumulative radiation doses in children with spinal dysraphism calculated mean childhood cumulative ED of 23 mSv with an additional cancer risk of 0.37 % (1 in 270) based on a risk of 16 % per Sv [10]. Therefore, the lowest dose investigation that meets clinical need should be used, particularly in patients where repeated exposures are required.

Densitometric vertebral fracture assessment (VFA) was first described by Genant in 2000 [11, 12]. There is a range of favourable VFA literature in adults [13–16], demonstrating sensitivity and specificity ranging from 62 to 97 % and 94 to 99 % respectively [14, 15, 17–22]. VFA is recommended as a complement to densitometry for improved clinical evaluation of asymptomatic VF in adults [23–25]. Although the importance of VF in the definition of osteoporosis in children is well established [26] and despite VFA being associated with lower radiation doses of 3–20 μSv [23, 27, 28] compared to 600–3000 μSv for radiographs [23, 27, 28], there are no recommendations for VFA in children. Generally, children with suspected reduced bone mineral density (BMD) have dual energy x-ray absorptiometry (DXA) to assess BMD and radiographs to identify vertebral fractures (VF), leading to significant lifetime cumulative radiation dose.

The aim of this study was to determine whether DXA, specifically iDXA (GE Healthcare Lunar iDXA, Buckinghamshire, UK), can replace radiographs for diagnosis of VF in children with suspected reduced BMD either with primary osteoporosis such as osteogenesis imperfecta or with secondary osteoporosis such as those treated with steroids or who have leukaemia.

## Methods

The study was funded by the National Institute for Health Research “Research for Patient Benefit Programme” (Reference PB-PG-0110-21240). Local ethics committee and Research and Development approval (Reference 11/YH/0292) and patient/guardian assent/consent were obtained.

**Fig. 1:**
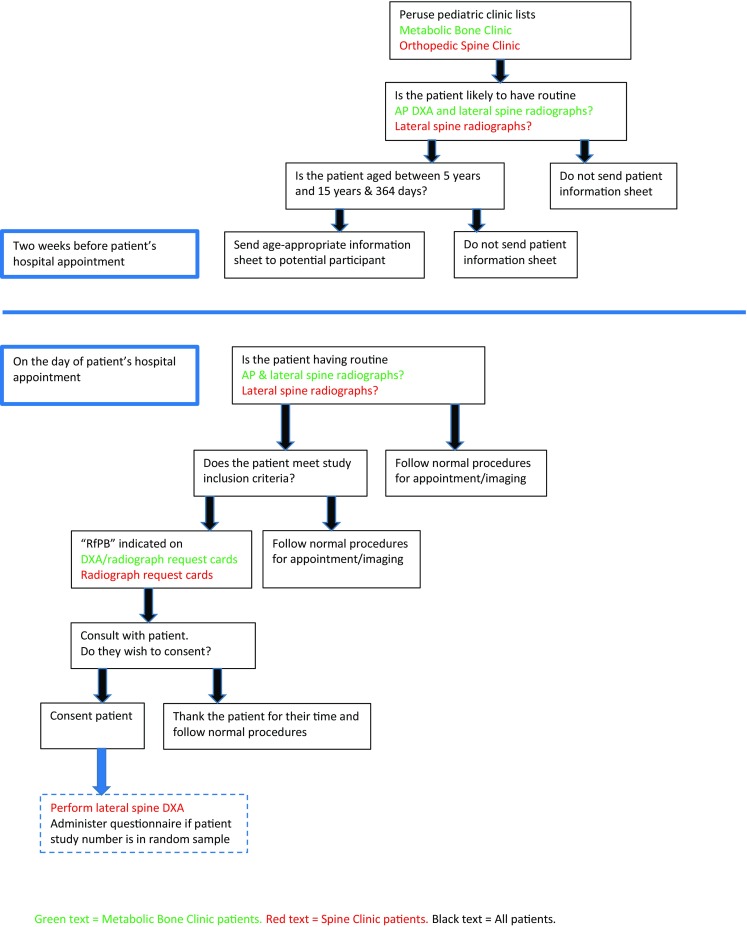
Flow chart demonstrating patient recruitment process from metabolic bone and spine clinics

Two hundred and fifty patients aged 5 years to 15 years (inclusive) were recruited between November 2011 and February 2014 from two tertiary paediatric centres; 200 with suspected reduced BMD attending the metabolic bone clinic for iDXA and lateral spine radiographs and 50 attending spine clinic requiring lateral spine radiographs as part of routine care who were consented for an additional lateral iDXA. Participants were only recruited into the study once (Fig. 1).

One hundred and fifty one patients were recruited prospectively and 99 retrospectively (33 from our centre, 66 from Birmingham Children’s Hospital - BCH).

Assuming (1) the true VF rate is 30 % and (2) 80 % sensitivity/specificity for the tests, then recruiting 250 patients (75 with VF), we can estimate sensitivity/specificity of DXA (±9 %) and radiography (±6 %) with 95 % confidence.

iDXA was performed according to published recommendations [29]. Radiographs were obtained on one of two local machines (TH3 Digital or TH Bucky Diagnost, Phillips, Guildford UK) or one of two machines at BCH (Luminus DRF, Siemens, Camberley UK, or CPI Wolverson Acroma unit, Willenhall UK) adhering to the European guidelines for spine radiographs in children [30]. Depending on patient size, single thoracolumbar or separate thoracic and lumbar exposures were taken. Radiographs were obtained in the lateral decubitus position for patients with suspected reduced BMD and in a standing lateral position for spine clinic patients. Average exposures were 73 kV, 82 kV and 103 kV for thoracic, lumbar and thoracolumbar radiographs respectively. Detector focus distance was 100 cm for decubitus and 210 cm for standing spine radiographs.

iDXA and radiographs for each patient were acquired on the same day.

Blinded to clinical information and corresponding results of the other modality, three consultant paediatric musculoskeletal radiologists (PB, IL, ACO), each with minimum 10 years’ experience, independently scored anonymised images in random order, for (1) presence of fractures and (2) image quality according to modified European criteria [30]. A hundred randomly selected pairs of images were read a second time. A final consensus read of all 250 radiographs acted as reference standard. Quantitative measurements using workstation measurement tools only took place at the reader’s discretion. The vertebrae were graded for fracture from 0 to 4 according to the simplified algorithm-based qualitative score (which is a modification of the Ferrar et al. algorithm-based qualitative vertebral fracture assessment technique [18]):

0) Normal
1) Fracture with 24 % or less height loss
2) Fracture with 25 % or more height loss
3) Non-osteoporotic deformity
4) Uncertain or unable to determine due to quality [31].

Because only lateral images were assessed and for consistency of vertebral level assignment between observers, the first vertebral body not associated with ribs was always designated L1 and the lowermost vertebral body associated with ribs was designated T12. If T12 and L1 could not be identified (e.g. excessive coning), all vertebrae were scored unreadable.

A questionnaire (non-validated) was randomly administered to assess patient and carer experience.

Radiation dose was calculated using dose area product (DAP) for radiographs and recorded exposure factors, scan areas and entrance surface dose (ESD) for iDXA. Average DAP was calculated and used to estimate average ED using PCXMC 2.0 software for different age groups to estimate the relative risk of each modality. Average lifetime additional cancer risk was calculated using the Health Protection Agency’s proposed total lifetime cancer risk per unit of ED (percentage per Sievert) as a function of age at exposure and sex.

Statistical analysis was performed using R Software Version 3.0.2 for PC. Using the consensus radiographic read as reference standard, we calculated and compared the prevalence of VF (percentage patients identified with one or more VF and percentage VF from the total of 3250 vertebrae) and iDXA/radiograph sensitivity/specificity. Previously surveyed clinicians initiate treatment once there is vertebral body height loss of 25 % or more plus pain [31]; therefore, patients were classified into two groups: no treatment (no VF or VF with a height loss of less than 25 %, VF_0_/VF_-25_) and treatment (one or more VF with a height loss of equal to/more than 25 %, VF_+25_) groups. Unreadable vertebrae within these groups were included in statistical analyses. Kappa statistics were used to assess inter/intraobserver and intermodality agreement. Fleiss’ kappa was used to assess agreement between all three observers simultaneously. Paired samples Student’s *t* test was used to compare radiation doses of the two modalities.

## Results

### Demographics

Mean patient age was 11.5 years; 104 (42 %) were male; 142 (57 %) self-classified as Caucasian, 109 (44 %) had osteogenesis imperfecta (OI). The other 90 children with suspected reduction in BMD had various diagnoses including inflammatory bowel disease, rheumatological conditions, coeliac disease, cystic fibrosis and unexplained fractures. 37(74 %) of the 50 spine clinic patients attended for scoliosis.

### Fracture characteristics/image analysis

#### Vertebral level

Of the 3250 vertebrae assessed, 364 (11 %) were fractured, with T7 being the most frequently fractured level (47/250, 19 %). Table 1 summarises fracture characteristics for the consensus and individual iDXA/radiograph reads.

**Table 1:** Summary of fracture characteristics for the 250 individual and consensus reads

	Reference Standard Consensus Radiograph		Observer 1 Radiograph		Observer 2 Radiograph		Observer 3 Radiograph		Observer 1 DXA		Observer 2 DXA		Observer 3 DXA	
	No.	%	No.	%	No.	%	No.	%S	No.	%	No.	%	No.	%
Total number of fractures	364	11	283	9	406	12	734	23	220	7	264	8	880	27
Most fractured level	T7(47)	(19)	T7(30)	(11)	L2(46)	(11)	T6(76)	(10)	T7, L3(24)	(11)	T7(31)	(12)	T7(93)	(11)
Number of fractures involving both endplates	163	45	165^a^	58	351	86	231^b^	31	101	46	197	75	188	21
Number of fractures involving one endplate	201	55	116 ^a^	41	55	14	502^b^	68	119	54	67	25	692	79
Number of fractures with height loss < 25 %	294	81	208	73	333	82	663	90	170	77	233	88	792	90
Number of patients with ≥ 1 fracture	90	36	78	31	95	38	159	64	71	28	82	33	176	70
Number of patients with ≥ 1 fracture and height loss < 25 %	87	35	73	29	92	37	156	62	66	26	80	32	171	68
Number of patients with ≥ 1 fracture and height loss ≥ 25 %	27	11	32	13	19	8	24	10	23	9	16	6	34	14
Number of patients with ≥ 1 fracture and both endplates affected	56	22	51	20	82	33	82	33	43	17	64	26	71	28
Number of patients with ≥ 1 fracture and one end plate affected	80	32	58	23	35	14	146	58	58	23	39	16	171	68
Number of unreadables	460	14	300	9	411	13	504	16	262	8	337	10	232	7

^a^two fractures were coded as having normal end plates; ^b^ one fracture had a missing (NA) endplate code

**Fig. 2:**
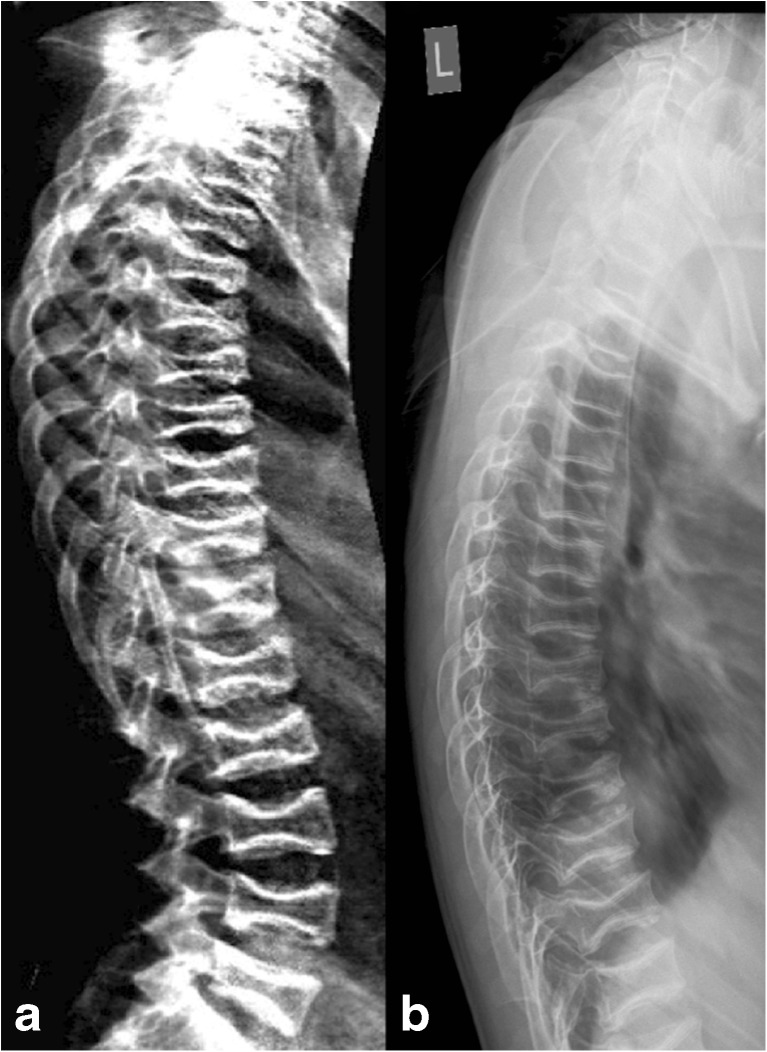
Lateral iDXA (**a**) and thoracic spine radiograph* (**b**) of patient 185, an 11-year-old female with osteogenesis imperfecta. Vertebrae T5 to T11 were independently identified by all observers on both iDXA and radiographic images as 2c fractures which translates to a height loss of more than or equal to 25 % (2), affecting both endplates (**c**). *The lumbar spine was included in the original radiographic examination, but for the illustrative purposes of this article, it has been omitted

Figure 2 compares (a) iDXA to (b) radiography in a patient with OI; vertebrae T5 to T11 were independently identified by all observers on both iDXA and radiography as fractures with a height loss equal to or more than 25 %.

**Fig. 3:**
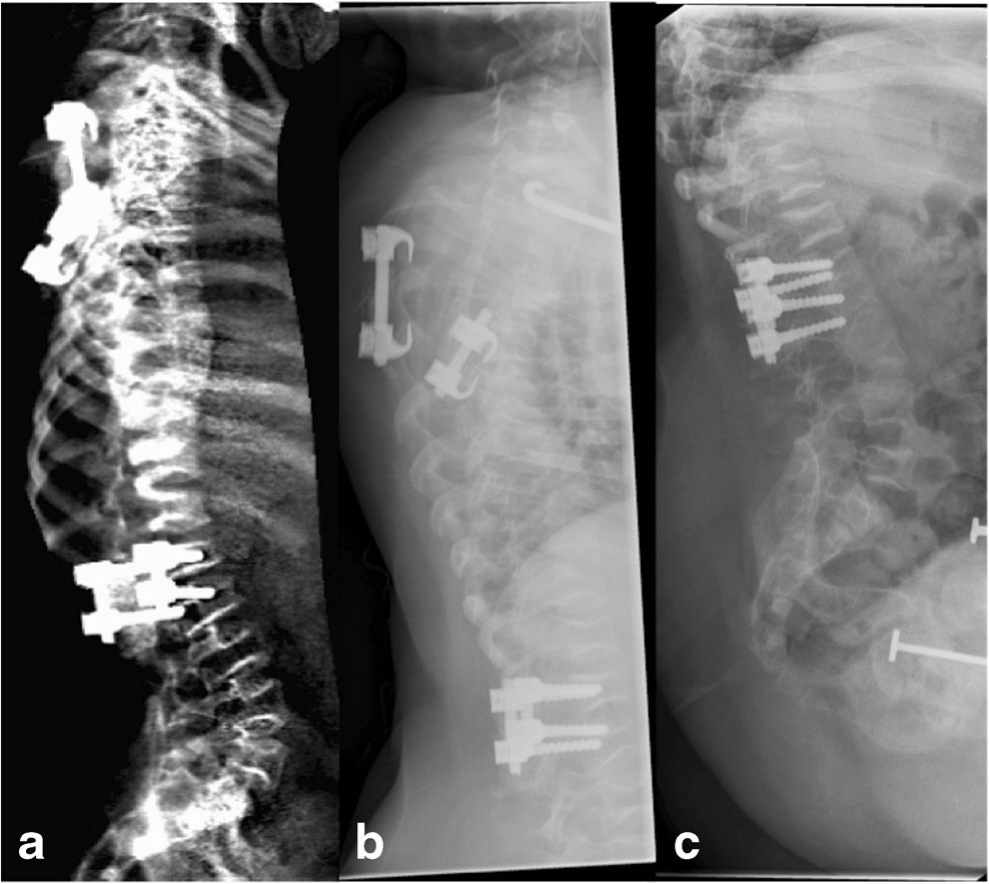
Lateral iDXA (**a**), thoracic spine radiograph (**b**) and lumbar spine radiograph (**c**) of patient 131, a 9-year-old female with osteogenesis imperfecta. The patient had severe multilevel fractures secondary to severe disease with resultant kyphoscoliosis degrading image quality on both iDXA and radiographs. On the consensus radiographic read T4 to T10 were graded as unreadable because of poor image quality

Figure 3 compares (a) iDXA to (b, c) radiography in a patient with severe OI.

##### Image quality

A total of 460 (14 %) vertebrae were unreadable. Reasons included excessive coning either obscuring T12/L1 so that reliable vertebral levels could not be assigned or obscuring other vertebrae, poor image quality and patient positioning.

Of the 3250 vertebrae, the number unreadable on iDXA was 262 (8 %), 337 (10 %) and 232 (7 %) for radiologists 1, 2 and 3 respectively. The number for radiographs was 300 (9 %), 411 (13 %) and 504 (16 %). The percentage of unreadable images varied by vertebral level, image modality and observer. Overall, the level with the highest number of unreadable vertebrae was T4 (27.6 %); this was true for all three observers and both modalities. Similarly, overall, the levels with the lowest number of unreadable vertebrae were L1 to L3 (4.8 %) and this was generally true for all three observers and both modalities. Results for each level, observer and modality are summarised in Table 2.

**Table 2:** Percentage of unreadable vertebral bodies for each vertebral level, image modality and observer

	Consensus	Observer 1	Observer 1	Observer 2	Observer 2	Observer 3	Observer 3	
	X-ray	X-Ray	DXA	X-Ray	DXA	X-Ray	DXA	
Vertebra	% unreadable	% unreadable	% unreadable	% unreadable	% unreadable	% unreadable	% unreadable	No. of cases
T4	27.6	20.8	16.4	20.4	19.6	30.4	16.8	250
T5	24.8	18.4	13.2	19.2	16.4	28.8	10.8	250
T6	22.4	14.8	10.4	18.8	14.0	27.2	7.2	250
T7	21.2	12.8	8.4	17.6	12.0	25.6	7.2	250
T8	18.8	10.0	8.0	15.2	9.6	19.6	6.4	250
T9	14.8	9.2	6.8	14.0	10.4	16.4	6.0	250
T10	14.8	8.4	6.4	13.2	10.0	14.4	7.6	250
T11	11.2	8.0	7.2	12.0	10.0	12.0	6.4	250
T12	8.4	6.0	6.8	10.0	8.4	7.2	5.6	250
L1	4.8	3.2	5.6	6.4	6.0	5.6	4.4	250
L2	4.8	2.8	5.2	6.0	6.0	4.0	4.4	250
L3	4.8	2.8	5.2	5.6	6.4	4.4	4.8	250
L4	5.6	2.8	5.2	6.0	6.0	6.0	5.2	250

Twenty-four patients had spinal rods in situ for scoliosis correction. There were on average less unreadable vertebrae for patients with spinal rods from iDXA (4, 7 and 4 for radiologists 1, 2 and 3 respectively) compared to radiographs (6, 8 and 7). The difference was statistically significant for radiologists 1 and 3 (*p* values 0.041 and 0.005 respectively).

**Fig. 4:**
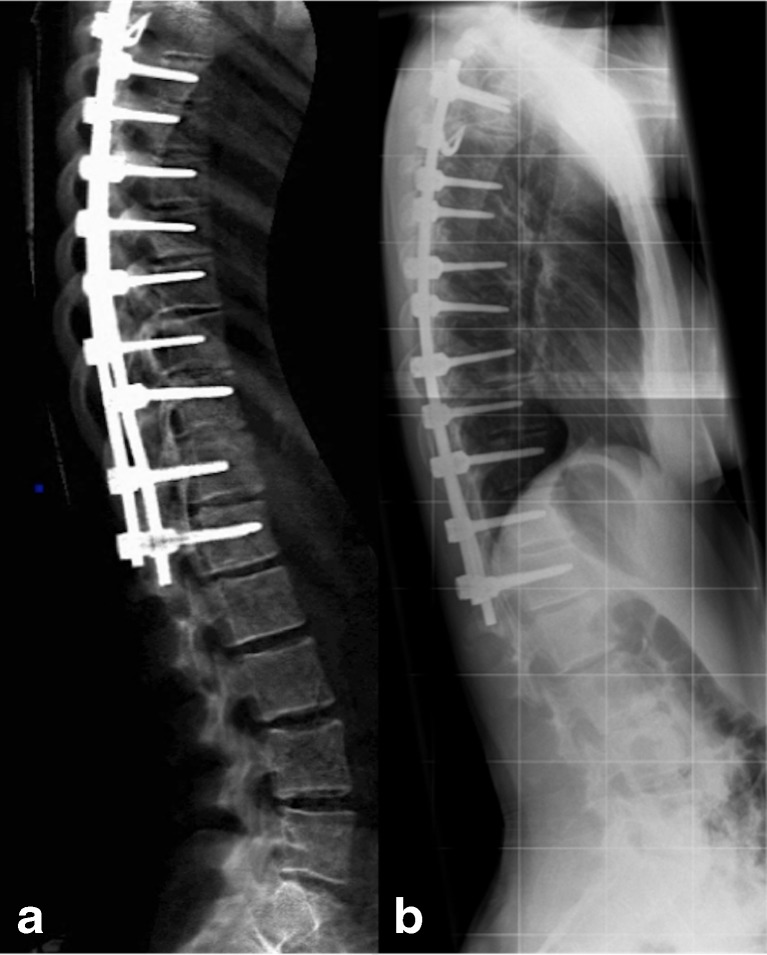
Lateral iDXA (**a**) and thoracic spine radiograph (**b**) of patient 80, a 14-year-old female with adolescent idiopathic scoliosis and previous spinal fixation. All observers independently scored vertebrae T4 to T6 as not fractured on iDXA. All observers were independently unable to score T4 to T6 on radiography because of poor image quality

Figure 4 compares (a) iDXA to (b) radiography in a postoperative scoliosis patient with spinal fixation; image quality with spinal rods in situ was degraded on radiographs from T4 to T6 but maintained on iDXA.

#### Patient level

Overall, 90 (36 %) patients had one or more VF (vertebral height loss 10 % or more). A total of 181 (72 %) patients had valid consensus radiograph data allowing definitive categorisation into no treatment (VF_0_/VF_-25_) or treatment (VF_+25_) groups. The remaining 69 (28 %) had a combination of unreadable vertebrae and VF_0_ and were excluded from diagnostic accuracy calculations as a result of the inability to give a definitive diagnosis (some or all of the unreadable vertebrae may have had significant loss of height).

Table 3 summarises diagnostic accuracy. On a patient level, for the diagnosis of any grade VF, iDXA had average sensitivity and specificity across the three radiologists of 78 % (95 % confidence interval (CI) 57–99 %) and 72 % (95 % CI 46–99 %) respectively and radiographs 84 % (95 % CI 70–99 %) and 72 % (95 % CI 47–97 %). For the diagnosis of VF_+25_, iDXA had average sensitivity and specificity across the three radiologists of 70 % (95 % CI 58–82 %) and 97 % (95 % CI 94–100 %) respectively and radiographs 74 % (95 % CI 55–93 %) and 96 % (95 % CI 95–98 %).

**Table 3:** Contingency table showing diagnostic accuracy of iDXA compared to reference standard

	iDXA								Radiograph							
	Any grade fracture				Fracture height loss ≥25%				Any grade fracture				Fracture height loss ≥25%			
	Consensus Reference standards Radiograph				Consensus Reference Standard Radiograph				Consensus Reference Standard Radiograph				Consensus Reference Standard Radiograph			
		Y	N	Total		Y	N	Total		Y	N	Total		Y	N	Total
Observer 1	Y	52	12	64	Y	18	2	20	Y	59	14	73	Y	23	7	30
	N	31	80	111	N	7	135	142	N	24	87	111	N	3	142	145
	Total	83	92	175	Total	25	137	162	Total	83	101	184	Total	26	149	175
		Sensitivity (95% CI)	Specificity (95% CI)			Sensitivity (95% CI)	Specificity (95% CI)			Sensitivity (95% CI)	Specificity (95% CI)			Sensitivity (95% CI)	Specificity (95% CI)	
		63% (51–73)	87% (78–93)			72% (51–88)	99% (95–100)			71% (60–81)	86% (78–92)			88% (70–98)	95% (91–98)	
Observer 2	Y	60	15	75	Y	14	2	16	Y	71	16	87	Y	15	3	18
	N	23	84	107	N	10	138	148	N	13	83	96	N	12	144	156
	Total	83	99	182	Total	24	140	164	Total	84	99	183	Total	27	147	174
		Sensitivity (95% CI)	Specificity (95% CI)			Sensitivity (95% CI)	Specificity (95% CI)			Sensitivity (95% CI)	Specificity (95% CI)			Sensitivity (95% CI)	Specificity (95% CI)	
		72% (61–82)	85% (76–96)			58% (37–78)	99% (95–100)			85% (75–91)	84% (75–90)			56% (35–75)	98% (94–100)	
Observer 3	Y	87	54	141	Y	19	8	27	Y	83	47	130	Y	18	5	23
	N	1	45	46	N	5	127	132	N	3	42	45	N	5	124	129
	Total	88	99	187	Total	24	135	159	Total	86	89	175	Total	23	129	152
		Sensitivity (95% CI)	Specificity (95% CI)			Sensitivity (95% CI)	Specificity (95% CI)			Sensitivity (95% CI)	Specificity (95% CI)			Sensitivity (95% CI)	Specificity (95% CI)	
		99% (94–100)	45% (35–56)			79% (58–93)	94% (89–97)			97% (90–99)	47% (37–58)			78% (56–93)	96% (91–99)	

Table 4 summarises the inter- and intraobserver agreement for the three observers for DXA and radiographs.

**Table 4:** Summary of observer agreements

Inter-observer agreement (*n* = 250)										
			DXA				Radiographs			
			Kappa			% agreement	Kappa			% agreement
			Mean	Min	Max	Mean	Mean	Min	Max	Mean
Fracture detection	Observers	1 vs 2	0.50	0.35	0.60	91	0.46	0.32	0.59	77
		1 vs 3	0.32	0.19	0.42	73	0.43	0.26	0.62	74
		2 vs 3	0.37	0.30	0.47	74	0.39	0.26	0.60	72
	Simultaneous agreement across 3		Fleiss’ Kappa			% agreement	Fleiss’ Kappa			% agreement
	observers		0.37			66	0.42			64
			Kappa			% agreement	Kappa			% agreement
			Mean	Min	Max	Mean	Mean	Min	Max	Mean
ABQ grading (1-4)	Observers	1 vs 2	0.47	0.35	0.56	84	0.49	0.39	0.62	82
		1 vs 3	0.30	0.19	0.40	70	0.34	0.27	0.41	70
		2 vs 3	0.35	0.30	0.43	72	0.40	0.32	0.46	71
	Simultaneous agreement across 3		Fleiss’ Kappa			% agreement	Fleiss’ Kappa			% agreement
	observers		0.351			64	0.400			62
Endplate assessment*			Kappa			% agreement	Kappa			% agreement
			Mean	Min	Max	Mean	Mean	Min	Max	Mean
	Observers	1 vs 2	0.44	0.33	0.56	83	0.49	0.41	0.62	81
		1 vs 3	0.29	0.16	0.38	69	0.33	0.25	0.62	69
		2 vs 3	0.33	0.25	0.41	70	0.38	0.31	0.44	69
	Simultaneous agreement across 3		Fleiss’ Kappa			% agreement	Fleiss’ Kappa			% agreement
	Observers		0.33			63	0.38			62
Intra-observer agreement (*n* = 100)										
			DXA				Radiographs			
			Kappa			% agreement	Kappa			% agreement
			Mean	Min	Max	Mean	Mean	Min	Max	Mean
Fracture detection	Observers	1	0.61	0.53	0.71	89	0.69	0.59	0.84	89
		2	0.69	0.58	0.78	89	0.68	0.57	0.80	84
		3	0.59	0.49	0.69	79	0.49	0.33	0.66	73
		All	0.63	0.49	0.78	86	0.62	0.33	0.84	82
			Kappa			% agreement	Kappa			% agreement
			Mean	Min	Max	Mean	Mean	Min	Max	Mean
ABQ grading (1-4)	Observers	1	0.58	0.43	0.67	87	0.64	0.51	0.70	86
		2	0.67	0.58	0.77	88	0.68	0.58	0.79	84
		3	0.56	0.47	0.68	76	0.48	0.32	0.65	72
		All	0.60	0.43	0.77	84	0.60	0.32	0.79	81
			Kappa			% agreement	Kappa			% agreement
			Mean	Min	Max	Mean	Mean	Min	Max	Mean
Endplate assessment*	Observers	1	0.58	0.45	0.68	88	0.64	0.54	0.71	86
		2	0.67	0.58	0.80	88	0.65	0.55	0.77	83
		3	0.54	0.47	0.62	75	0.46	0.32	0.63	70
		All	0.60	0.45	0.80	83	0.58	0.32	0.77	80

*Missing values recorded as not applicable

Table 5 summarises intermodality agreement between the three observers for iDXA versus radiographs, iDXA versus consensus/reference standard radiograph and radiograph versus consensus/reference standard radiograph.

**Table 5:** Summary of intermodality agreements

iDXA and radiographs (*n* = 250)						
			Kappa			% agreement
			Mean	Min	Max	Mean
Fracture detection	Observers	1	0.41	0.32	0.50	83
		2	0.45	0.38	0.57	80
		3	0.39	0.29	0.54	68
		All	0.42	0.29	0.66	77
			Kappa			% agreement
			Mean	Min	Max	Mean
ABQ grading (1-4)	Observers	1	0.37	0.31	0.46	81
		2	0.43	0.35	0.55	79
		3	0.38	0.29	0.50	67
		All	0.39	0.29	0.55	75
			Kappa			% agreement
			Mean	Min	Max	Mean
Endplate assessment	Observers	1	0.36	0.29	0.46	80
		2	0.43	0.37	0.50	79
		3	0.33	0.26	0.43	64
		All	0.37	0.26	0.50	74
iDXA and consensus radiographs (*n* = 250)						
			Kappa			% agreement
			Mean	Min	Max	Mean
Fracture detection	Observers	1	0.32	0.21	0.45	76
		2	0.39	0.33	0.44	78
		3	0.34	0.23	0.51	69
		All	0.35	0.21	0.51	74
			Kappa			% agreement
			Mean	Min	Max	Mean
ABQ grading (1-4)	Observers	1	0.30	0.21	0.40	74
		2	0.38	0.30	0.42	77
		3	0.33	0.24	0.50	68
		All	0.33	0.21	0.50	73
			Kappa			% agreement
			Mean	Min	Max	Mean
Endplate assessment	Observers	1	0.28	0.18	0.41	74
		2	0.36	0.29	0.44	76
		3	0.31	0.23	0.47	67
		All	0.32	0.18	0.47	72
Radiographs and consensus radiographs (*n* = 250)						
			Kappa			% agreement
			Mean	Min	Max	Mean
Fracture detection	Observers	1	0.55	0.39	0.61	84
		2	0.55	0.45	0.63	82
		3	0.46	0.38	0.58	75
		All	0.52	0.38	0.63	81
			Kappa			% agreement
			Mean	Min	Max	Mean
ABQ grading (1-4)	Observers	1	0.53	0.40	0.61	82
		2	0.54	0.46	0.64	81
		3	0.46	0.37	0.57	74
		All	0.51	0.37	0.64	79
			Kappa			% agreement
			Mean	Min	Max	Mean
Endplate assessment	Observers	1	0.51	0.40	0.59	82
		2	0.50	0.39	0.60	79
		3	0.43	0.37	0.53	72
		All	0.48	0.37	0.60	78

#### Radiation dose

A total of 144 patients had valid radiation dose data; 95 (66 %) were male, mean age was 11.8 years (5–15 years) and mean weight was 41.1 kg (14.3–87.5 kg). The mean DAP for iDXA was 18.0 μGy/m^2^ (SD 3.4) compared to 64.4 μGy/m^2^ (SD 76.7) for radiographs, a difference of 46.4 μGy/m^2^ (95 % CI 33.7–59.1), *p* < 0.001. Average age-adjusted ED for iDXA was 41.9 μSv compared to 232.7 μSv for radiographs.

The average lifetime additional cancer risk per lateral iDXA was calculated to be 0.001 % and 0.000 % for patients aged 5–10 and 10–15 years respectively for both sexes. Per lateral spine radiograph the additional lifetime cancer risk was 0.003 % for boys and 0.002 % for girls aged 5–15 years.

#### Patient experience

Eighty-five sets (85 %) of patient/carer questionnaires were returned. Of these, 77 (91 %) were completed by patient and carer, five (6 %) by the carer only and three (3 %) by the child only. Of the 82 carers that completed a questionnaire, 11 (13 %) thought their child had difficulty staying still whilst the radiographs were obtained compared to 8 (10 %) for iDXA (*p* = 0.549). Two (2 %) carers thought their children (aged 10.3 and 15.8 years) found the noise of the iDXA upsetting or frightening.

Eighty children (32 aged 5–11 years and 48 aged 12–15 years) completed questionnaires. Thirty-nine (49 %) preferred iDXA while 27 % (34 %) had no preference. Sixty-nine (86 %) did not find moving about the hospital for the different tests unacceptable.

There were no adverse effects of either iDXA or radiographs.

## Discussion

This is the largest study to date assessing whether VFA can replace spine radiographs in children. Overall we found iDXA had similar sensitivity and specificity to radiography and good intraobserver agreement, on average higher than the intraobserver agreement of radiography. A similar study of VFA in children concluded that its utility was limited by compromised visibility and poor diagnostic accuracy [27]. However, those results were based on older DXA technology (Hologic Densitometer), a relatively small sample size (*n* = 65) and acquisition of DXA and radiographic images not on the same day but within 6 months of each other. A more recent comparative study using newer DXA technology (Hologic Discovery A Densitometer) reported sensitivity (96 %) and specificity (100 %) on a patient level (some vertebrae were excluded from analysis because of poor visibility) [32]. Another recent study of VFA in 165 children and adolescents compared 20 of the subjects’ VFA with lateral spine radiographs (obtained within 2 months of each other), reporting sensitivity of 83 % and specificity of 100 % for VFA [33]. This study did not assess T4 or T5 and again excluded unreadable vertebrae from statistical analyses [33]. Diagnostic accuracy of both studies [32, 33] was higher than ours for both DXA and radiographs; inclusion of poorly visualised vertebrae in our statistical analyses may be seen either as a weakness or strength. Whilst diagnostic accuracy will have been improved had we excluded all poor quality images, the data as presented demonstrates the worst-case scenario.

Our results indicate that iDXA had a (statistically insignificant) lower unreadable rate than radiographs (up to 16 % for both). These rates are similar to previous studies performed on adult (DXA and radiographs) [13, 14] and paediatric (radiographs) [34] populations. However iDXA had a (statistically significant) better image quality than radiographs when spinal rods were in situ.

DAP was chosen to estimate radiation dose because accurate ESD measurements using thermoluminescent dosimeters are challenging at low doses and more labour intensive. The radiographic systems had DAP meters installed and the iDXA system recorded scan area, offering simple methods for estimating doses in a large number of patients by only requiring the periodic measurement of ESD to ensure stability. Commonly published DXA doses relate to post-menopausal women over the age of 60 and reference dose data from 2006 [2]; the lifetime risk of fatal cancer in children is approximately four to five times [5] higher than this adult group. Published differences in radiation dose for radiographs and VFA (200:1) are higher than the differences shown by our study (5.5:1) [23, 27, 28]; however, published data commonly relates to standard DXA spine scans (ca. 10 cm × 20 cm) with a scan area of ca. 200 cm^2^, whereas the scans performed in this study had an average area of ca. 700 cm^2^, replicating conventional film coverage. This accounts for an estimated 3.5-fold increase in estimated ED. Our average ESD measurement of 235 μGy^2^ is similar to the published values of up to 352 μGy^2^ for a different manufacturer’s scanner (Hologic QDR 4500-A) [2]. The remainder of the difference is likely due to newer digital radiographic technology with significantly lower doses compared to previous non-digital technologies. Even though dose reduction was lower than expected (demonstrating the benefit of optimised exposures delivered by dedicated paediatric radiology departments), an average annual ED reduction of 232.7 μSv per patient amounts to a considerable childhood/lifetime cumulative dose reduction, particularly given the comparable diagnostic accuracy and patient/carer acceptability of VFA. Based on average dose calculations from our cohort of patients, for a female, estimated cumulative ED of at least 2097 μSv from an annual spine radiograph between the ages of 5 and 15 years would give an additional lifetime cancer risk of 0.022 % (1 in 4545). For a male, estimated cumulative ED would be 2930 μSv with an additional lifetime cancer risk of at least 0.033 % (1 in 3030). Although the overall risk per patient is low, total numbers of patients are relatively high and it is an avoidable risk without compromising diagnostic information.

If conventional radiography is required as a baseline to assess spinal deformity, such as scoliosis or kyphosis in this select group of patients with suspected reduction in BMD, then the use of EOS® for full standing radiographs of the spine is an alternative method of reducing cumulative radiation dose [35]. The limiting factor for the use of this alternative low dose technique is its availability. EOS systems are more expensive than conventional radiographic equipment and estimates of patient throughput at national level suggest that EOS is not cost-effective [36]. Therefore, the National Institute for Health and Care Excellence (NICE) does not currently recommend the routine use of EOS in the National Health Service (NHS) [37]. Although EOS produces images of equal or better quality than radiographs at doses comparable to DXA, it does not mitigate the need for BMD assessment and therefore a test that can simultaneously assess both in those children who do not have scoliosis/kyphosis is preferable.

The major limitations of this study (and others of diagnostic accuracy) relate to the lack of an objective gold standard. Firstly, because there is no agreed standardised objective method for the diagnosis of VF, we cannot be certain which prevalent fractures were truly fractures. We used the consensus radiographic read of three experienced observers as reference standard. Radiographic cone beam technology has the disadvantage of producing divergent x-ray beams causing parallax and distorting the shape of the vertebrae at the extremities of the radiograph. Conversely, the fan beam technology in DXA is perpendicular to each vertebral body as the source travels down the spinal column [27]. The parallax effect seen in radiographs may affect diagnostic accuracy, particularly for subtle fractures or normal physiological change in vertebral body shape and height. It is possible that mild fractures were over-called on radiographs rather than missed on iDXA. We accept that our selected reference standard may be imperfect, but it is at least as reliable as standards used in daily practice and is expected to be reliable for those vertebral fractures that would merit treatment (height loss greater than 25 %).

Secondly, the higher intermodality agreement of individual radiograph compared to individual iDXA reads is in part to be expected, because for individual and consensus radiographs we were scoring not only the same modality but also the same images. Despite this advantage, radiographs did not significantly outperform iDXA.

Thirdly, disadvantages of consensus scoring in general are well documented [38] and applicable to this study; however, inter- and intraobserver agreement for individual reads was similar for both iDXA and radiographs. Therefore, for any individual radiologist, clinical opinion and hence patient management would be the same irrespective of whether diagnosis of VF was made from DXA or from radiographs.

Finally, the use of conventional statistical methods for studies of diagnostic accuracy for which there is no gold standard has been questioned and more appropriate methodology suggested [39]. An interesting future study would be to apply some of these methodologies (e.g. latent class analysis) to our raw data.

In conclusion, diagnostic accuracy of iDXA and radiographs for the detection of VF in children are comparable; parents had no strong preference for either modality, whilst the majority of children either preferred iDXA or had no preference. Incidentally we demonstrated improved image quality of iDXA for scoliosis patients with in situ spinal rods. A single iDXA scan provides an average annual effective dose reduction of at least 232.7 μSv per patient. Given the large numbers of children at risk of VF (skeletal dysplasias, steroid therapy, anticancer treatment etc.) this amounts to considerable childhood and population lifetime cumulative dose reductions. In accordance with the principles of “as low as reasonably achievable” [40] and “image gently” [41], we believe that in children with suspected reduced BMD, either with primary osteoporosis such as osteogenesis imperfecta or with secondary osteoporosis such as those treated with steroids or who have leukaemia, DXA (using modern scanners) should replace conventional radiographs for the diagnosis of VF.

## Abbreviations and acronyms

BMD: bone mineral density
DAP: dose area product
DXA: dual energy x-ray absorptiometry
ED: effective dose
ESD: entrance surface dose
OI: osteogenesis imperfecta
sABQ: simplified algorithm-based qualitative
VF: vertebral fracture(s)
VFA: densitometric vertebral fracture assessment
